# Pain Among Cancer Survivors

**DOI:** 10.5888/pcd17.190367

**Published:** 2020-07-09

**Authors:** M. Shayne Gallaway, Julie S. Townsend, Daniel Shelby, Mary C. Puckett

**Affiliations:** 1 Centers for Disease Control and Prevention, National Center for Chronic Disease Prevention and Health Promotion, Division of Cancer Prevention and Control, Atlanta, Georgia; 2 Centers for Disease Control and Prevention, National Center for Injury Prevention and Control, Division of Violence Prevention, Atlanta, Georgia

## Abstract

**Introduction:**

Pain is one of the most common symptoms that people with cancer experience. Identification of demographic, physiologic, and behavioral correlates of pain among cancer survivors could help identify subgroups most in need of pain management.

**Methods:**

We analyzed data from the 2012, 2014, and 2016 Behavioral Risk Factor Surveillance System Cancer Survivorship Optional Module, which was completed by 18 states and territories, to describe demographic and physiologic characteristics of cancer survivors reporting physical pain caused by cancer or cancer treatment. Adjusted and unadjusted population-based estimates and 95% confidence intervals were calculated.

**Results:**

Of 12,019 cancer survivor respondents, 9.5% reported current pain related to cancer or cancer treatment. Current pain differed significantly by sex, race/ethnicity, age, and cancer type. Current pain was reported most often among survivors with more than 3 chronic diseases (16.7%) compared with survivors with none (8.1%) or 1 or 2 (10.0%). Pain was higher among survivors reporting fair or poor general health (18.0%) than among survivors reporting otherwise, and higher among survivors reporting more than 14 days of poor physical health (16.6%) or poor mental health (14.8%) compared with less than 14 days (in the past 30 days).

**Conclusions:**

Our results suggest that approximately 10% of cancer survivors in the United States are experiencing pain that may have persisted for years after their initial diagnosis and may not be adequately controlled. Increasing knowledge of the most appropriate pain management planning and strategies for controlling short- and long-term chronic pain among cancer survivors could help reduce the prevalence of pain.

**Summary**

**What is already known about this topic?**

Pain is one of the most common symptoms that cancer survivors experience.

**What is added by this report?**

We investigated the demographic and physiologic characteristics of cancer survivors who experience pain.

**What are the implications for public health practice?**

Knowledge of the demographic and physiologic characteristics of cancer survivors most likely to report experiencing cancer or cancer treatment–related pain can help educate clinicians, cancer survivors, and caregivers and inform regular screening for and proper characterization of pain, pain treatment methods, and ongoing monitoring of treatment efficacy.

## Introduction

Approximately 15.5 million cancer survivors (people who received a diagnosis of cancer) were alive in the United States in 2016, and that number is expected to increase to nearly 20 million by 2026 (1,2) because nearly half of cancer survivors live longer than 10 years (1). Pain is one of the most common symptoms experienced among cancer patients and can be caused by cancer itself (eg, tumor pressing on nerves, bones, or organs), surgery, treatment and treatment side effects (eg, peripheral neuropathy, mouth sores, radiation mucositis), or other procedures and tests (3,4). Research suggests that pain occurs in approximately 20% to 50% of cancer survivors (4,5). Clinical factors that may be associated with survivor pain are the stage (type and invasiveness) of the tumor, type of anticancer treatment received, time since completing treatment, comorbid conditions, and initial pain management (3,6–8). Effective methods are available to prevent and control pain during and after cancer treatment, including early recognition of pain symptoms, characterization and communication about pain type and severity, pharmacologic and nonpharmacologic pain control options, and patient education to ensure adequate pain and symptom management through all phases of cancer treatment and following treatment (9–11). Although pain can be controlled, approximately 30% of cancer survivors do not receive pain medication proportional to their pain intensity (12). Pain can negatively affect a cancer survivor’s daily functional status and quality of life (7,8,13) and can persist for years. Cancer survivors may experience psychological distress when pain persists after completion of cancer treatment (4), and untreated pain can lead to unnecessary hospital admissions (14,15). Identification of demographic, physiologic, and behavioral correlates of pain in cancer survivors can provide important information on specific subgroups most in need of pain management.

Cancer survivors can suffer from both short- and long-term pain (3); however, treatment-related pain typically diminishes over time. Approximately 1 year after diagnosis, more than 90% of patients observed in the American Cancer Society’s Study of Cancer Survivors-I study reported short-term pain symptoms related to their cancer or its treatment (7); 6% of Australian adult cancer survivors reported pain intensity as “quite a bit/very much” 5 to 6 years post-diagnosis (8), and approximately 20% of childhood cancers survivors (with a mean survival time from diagnosis of 16.5 years) reported recent pain attributed to their previous cancer or cancer treatment (6). Pain may be more common among certain subpopulations, such as breast and lung cancer survivors, because of the cancer stage or surgery received (14,16). The prevalence and severity of chronic pain among cancer survivors has also been shown to vary by racial populations (ie, pain severity reported among blacks is greater than among whites) and sex (ie, occurrence of pain reported among females is greater than among males) (13).

Pain can also be associated with other physiologic symptoms (13,17). Cancer survivors who report pain also report lack of sleep, fatigue, and mental health issues (13,17). Patients with comorbid conditions may have significantly greater physical functional pain and associated limitations and may be less likely to improve with standard pain management (9).

Despite all the evidence related to the prevalence of pain and comorbidities with pain and the availability of effective pain management strategies, cancer survivors may not be fully aware of the long-term prevalence of cancer-related pain that may persist after treatment completion. Thus, behaviors associated with pain need to be better characterized to help inform clinicians treating cancer survivor populations that could most benefit from additional education, resources, and strategies to manage cancer-related chronic pain. To this end, the purpose of our study was to use the most current national data to describe demographic and physiologic characteristics of cancer survivors who reported physical pain caused by cancer or cancer treatment. Informing patients and providers will aid in promoting collaborative relationships critical to providing optimal pain management.

## Methods

### Survey

We analyzed cross-sectional data from the 2012, 2014, and 2016 Behavioral Risk Factor Surveillance System (BRFSS), a representative, state-based telephone survey that recruits residents via landline or cellular telephone (18). We used data from states and territories that administered the Cancer Survivorship Optional Module (Alabama, Alaska, Georgia, Hawaii, Idaho, Iowa, Indiana, Kansas, Louisiana, Michigan, Mississippi, Missouri, Nebraska, Ohio, South Dakota, Virgin Islands, Vermont, and Wisconsin). The median response rates by year among states with the survivorship module were 50.1% (2012), 50.2% (2014), and 51.4% (2016) and were representative of the populations surveyed (18). Respondents were asked if they had ever been told by a doctor, nurse, or other health care professional that they had cancer. If they answered yes, they were asked which type or how many different types of cancer they had and their age at first diagnosis. We excluded cancer survivors who reported nonmelanoma skin cancer, were unsure of their cancer type, or refused to provide a response to these questions.

### Pain and demographic characteristics

We compared demographic and physiologic characteristics among all cancer survivors who did and did not report cancer pain or cancer treatment–related pain. To assess cancer pain and cancer treatment–related pain, respondents were asked, “Do you currently have physical pain caused by your cancer or cancer treatment?” Those who answered yes were classified as experiencing physical pain, regardless of when their most recent cancer diagnosis was. They were also asked, “Is your pain currently under control?” Demographics assessed were sex; age (18–39, 40–49, 50–65, ≥65 ); race/ethnicity (non-Hispanic white, non-Hispanic black, other (Asian, American Indian, Alaskan Native, Pacific Islander, other, Hispanic [of any race]); education (less than high school diploma, high school diploma or general education diploma (GED), some college [no degree or an associate degree], college degree [undergraduate degree, graduate degree]); current health insurance (yes or no); currently employed (yes or no); and whether medical care costs have restricted care (yes or no). We calculated and categorized years since diagnosis by using the respondents’ current age and age at first cancer diagnosis (≤5, 6–10, >10).

### Physiologic characteristics

Respondents were asked to describe their general health as either excellent, very good, good, fair, or poor. They were also asked how many days in the past month (past 30 days) their physical health and mental health were good or better and how many days they considered their general health as poor. They reported the following behaviors for the month preceding the survey: average number of hours of sleep per night (subsequently categorized as <7 h or ≥7 h), smoking status (yes or no), binge drinking (yes or no), and co-occurring chronic diseases (arthritis, asthma, chronic obstructive pulmonary disease, depression, diabetes, heart disease, heart attack, kidney problems, stroke). We compared the proportion of respondents experiencing physical pain who described their general health as fair or poor (vs good, very good, or excellent) and also who reported 14 or more days (vs fewer than 14 days) of poor general health, mental health, or physical health.

### Statistical analysis

We used SAS/SUDAAN version 11.0.1 (Research Triangle Institute) statistical software for analyses to account for the complex BRFSS design. Kansas, Nebraska, and Ohio elected to administer the survivorship module on a subset of their sample, so their survey weights accounted for this split survey design. Survey weights were also adjusted for states included in multiple years (Missouri, Nebraska, and Wisconsin). All estimates were weighted to provide population-based estimates, and 95% confidence intervals (CIs) were generated; 95% CIs are presented to allow for informal comparisons among prevalence estimates, without specifying a referent group. To determine how pain affects health status, significant associations between pain and health status (general, physical, and mental), adjusted prevalence estimates (ie, predicted marginals) were generated from multivariable logistic regression models adjusted for demographic and clinical factors. Best-fit models were determined through backward deletion of least significant variables. Given there was no human subject contact during completion of these secondary data analyses, institutional review board approval was not required.

## Results

Of the 12,019 cancer survivor respondents (total weighted sample N = 2,949,032) who participated in the BRFSS during the study period, 9.5% (n = 1,146) reported experiencing pain related to cancer or cancer treatment. Female cancer survivors (12.5%) reported more physical pain related to cancer treatment than males (8.9%), and non-Hispanic black cancer survivors (22.9%) reported more physical pain related to cancer treatment than non-Hispanic whites (10.0%) and other racial/ethnic populations (11.2%) (Table 1). Cancer survivors aged 65 or older (6.2%) reported less pain than survivors younger than 65 (13.6%–20.3%). Survivors who were out of work or unable to work were also more likely to report physical pain related to cancer treatment (23.9%) than cancer survivors who were employed (11.5%), retired (5.8%), or reported another employment status (13.8%). Physical pain related to cancer treatment did not differ substantially among cancer survivors without current health insurance (15.2%) and survivors with current health insurance (10.8%); however, cancer survivors who reported that medical costs restricted their health care were more likely to report current pain (21.6%) related to cancer treatment than those who did not report health care restrictions because of medical costs (9.8%).

**Table 1:** Demographic Characteristics Among Cancer Survivors With (n = 1,146) and Without (n = 10,873) Current Pain Related to Cancer or Cancer Treatment, Behavioral Risk Factor Surveillance System, 2012–2016

Characteristic	Current Pain			No Current Pain		
	n^a^	N^b^	% (95% CI)^b^	n^a^	N^b^	% (95% CI)^b^
**Sex**						
Male	308	111,044	8.9 (7.2–11.0)	4,180	1,129,960	91.1 (89.0–93.1)
Female	838	213,899	12.5 (11.2–14.0)	6,693	1,494,129	87.5 (86.0–88.8)
**Age at interview, y**						
18–39	74	49,913	19.6 (13.4–27.7)	377	205,117	80.4 (72.3–86.6)
40–49	122	57,231	20.3 (15.2–26.6)	564	224,060	79.7 (73.4–84.8)
50–64	499	124,631	13.6 (11.9–15.5)	2,806	792,319	86.4 (84.5–88.1)
≥65	451	93,169	6.2 (5.3–7.2)	7,126	1,402,593	93.8 (92.8–94.7)
**Race/ethnicity**						
Non-Hispanic white	810	209,376	10.0 (8.9–11.2)	8,409	1,883,943	90.0 (88.8–91.1)
Non-Hispanic black	64	39,115	22.9 (15.4–32.6)	378	132,019	77.1 (67.4–84.6)
Other^c^	272	76,453	11.2 (9.1–13.6)	2,086	608,125	88.8 (86.4–90.9)
**Education**						
<High school diploma	101	52,573	15.7 (10.8–22.2)	751	283,208	84.3 (77.8–89.2)
High school diploma or GED	372	108,633	11.4 (9.7–13.4)	3,533	842,411	88.6 (86.6–90.3)
Some college	363	107,099	11.2 (9.6–13.0)	3,052	849,364	88.8 (87.0–90.4)
College degree	307	56,320	8.0 (6.5–9.8)	3,519	645,678	92.0 (90.2–93.5)
**Employment**						
Employed	367	115,127	11.5 (9.4–14.0)	3,220	885,430	88.5 (86.0–90.6)
Out of work/unable to work	327	104,661	23.9 (20.4–27.9)	1,064	332,679	76.1 (72.1–79.6)
Retired	367	73,905	5.8 (4.9–6.8)	5,904	1,209,568	94.2 (93.2–95.1)
Other	81	30,512	13.8 (9.6–19.5)	648	190,076	86.2 (80.5–90.4)
**Insurance**						
Yes	1,084	304,364	10.8 (9.7–12.0)	10,510	2,504,395	89.2 (88.0–90.3)
No	62	20,580	15.2 (10.5–21.6)	349	114,802	84.8 (78.4–89.5)
**Medical costs restrict care**						
Yes	207	66,627	21.6 (17.3–26.7)	756	241,217	78.4 (73.3–82.7)
No	937	257,861	9.8 (8.7–11.0)	10,103	2,378,923	90.2 (89.0–91.3)
**Pain under control**						
Yes, with medications	443	125,810	39.5 (34.0–45.3)	—	—	—
Yes, without medications	464	128,389	40.3 (35.1–45.8)	—	—	—
No, with medications	92	20,822	6.5 (4.7–9.0)	—	—	—
No, without medications	126	43,201	13.6 (10.4–17.5)	—	—	—

Abbreviations: —, not applicable; CI, confidence interval; GED, general education diploma.

a Unweighted number of respondents.

b Weighted population number and prevalence.

c Includes American Indian/Alaska Native, Hispanic, Pacific Islander, and other.

Survivors of lung cancer (28.3%), female breast cancer (19.1%), leukemia/lymphoma (18.0%), and colorectal cancer (15.7%) reported more physical pain related to cancer treatment than survivors with other cancers in our study (Table 2). Cancer survivors who were aged 65 or older at diagnosis were less likely to report physical pain (5.9%) related to treatment than survivors diagnosed when they were younger than 65 (10.1%–15.3%). Cancer survivors whose cancer was diagnosed more than 10 years in the past (9.5%) and survivors whose cancer was diagnosed 10 years or less in the past (11.9%­–13.0%) reported a similar prevalence of physical pain related to cancer treatment.

**Table 2:** Prevalence of Pain Reported by Cancer Survivors by Cancer Type, Behavioral Risk Factor Surveillance System, 2012–2016

Variable	Current Pain (n = 1,146)			No Current Pain (n = 10,873)		
	n^a^	N^b^	% (95% CI)^b^	n^a^	N^b^	% (95% CI)^b^
**Number of different cancer types**						
1	842	248,970	10.6 (9.4–11.9)	8,543	2,095,575	89.4 (88.1–90.6)
≥2	304	75,973	12.6 (10.2–15.5)	2,330	528,514	87.4 (84.5–89.8)
**Cancer type**						
Lung	62	18,117	28.3 (19.6–38.9)	211	45,900	71.7 (61.1–80.4)
Female breast	398	104,506	19.1 (16.3–22.4)	2,236	441,556	80.9 (77.6–83.7)
Leukemia/lymphoma	69	19,286	18.0 (12.6–25.1)	343	87,907	82.0 (74.9–87.4)
Colorectal	94	31,418	15.7 (10.0–23.7)	722	168,748	84.3 (76.3–90.0)
Gynecologic cancers not including cervix	51	10,108	7.4 (4.8–11.1)	575	127,318	92.6 (88.9–95.2)
Cervix	49	17,294	6.9 (4.3–10.7)	731	234,629	93.1 (89.3–95.7)
Prostate	66	20,522	5.3 (3.3–8.3)	1,394	368,240	94.7 (91.7–96.7)
Melanoma	58	16,262	2.7 (1.8- 4.1)	2,220	584,729	97.3 (95.9–98.2)
All other	278	82,986	14.0 (11.5–16.9)	2,131	511,203	86.0 (83.1–88.5)
**Age at diagnosis, y**						
18–39	253	108,828	15.3 (12.2–19.0)	1,872	604,380	84.7 (81.0–87.8)
40–49	251	67,143	14.2 (11.8–17.1)	1,548	405,217	85.8 (82.9–88.2)
50–64	449	105,164	10.1 (8.7–11.7)	3,937	938,236	89.9 (88.3–91.3)
≥65	171	37,932	5.9 (4.6–7.5)	3,200	607,504	94.1 (92.5–95.4)
**No. years since diagnosis**						
≤5	402	127,788	13.0 (10.9–15.4)	3,293	854,978	87.0 (84.6–89.1)
6–10	263	74,862	11.9 (9.7–14.6)	2,177	553,115	88.1 (85.4–90.3)
>10	455	115,346	9.5 (8.0–11.2)	4,835	1,100,442	90.5 (88.8–92.0)

Abbreviation: CI, confidence interval.

a Unweighted number of respondents.

b Weighted population number and prevalence.

**Figure 1:**
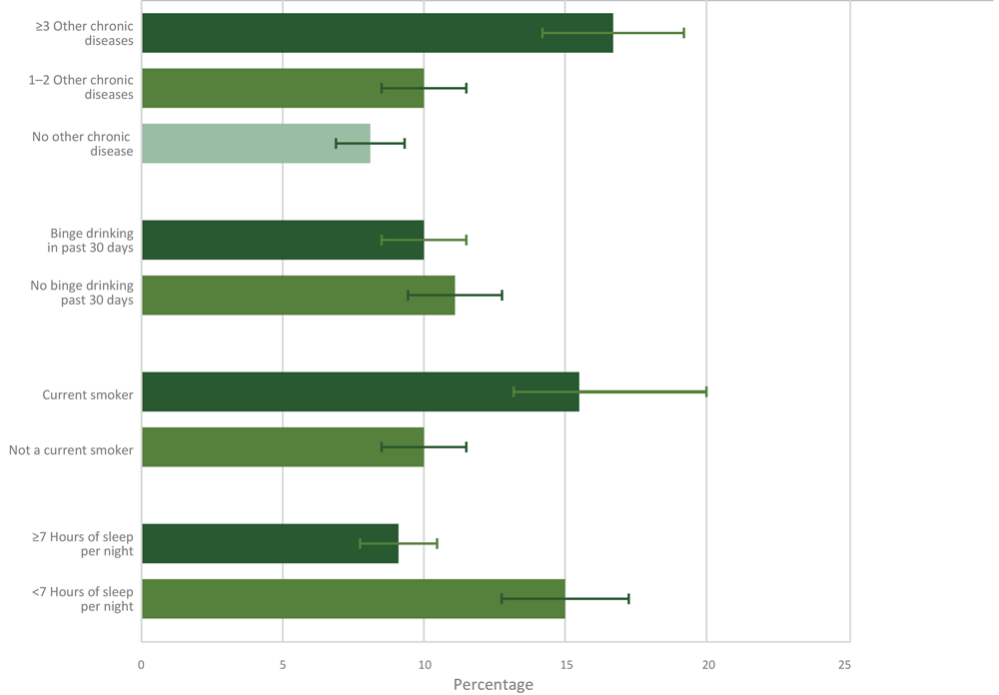
Prevalence and 95% confidence intervals of current pain related to cancer or cancer treatment by comorbid and behavioral characteristics. Data on hours of sleep per night among cancer survivors from the Behavioral Risk Factor Surveillance System were available only for 2014 and 2016 (n = 9,910). Other chronic diseases included were a history of arthritis, asthma, chronic obstructive pulmonary disease, depression, diabetes, heart attack, heart disease, kidney disease, and stroke. Brackets indicate confidence intervals.

Cancer survivors who reported 3 or more other chronic diseases (16.7%) reported more physical pain than survivors with none (8.1%) or 1 or 2 (10.0%) other chronic diseases (Figure 1). Current smokers (15.5%) also reported more physical pain than current nonsmokers (10.0%), and cancer survivors who reported sleeping less than 7 hours nightly (15.0%) reported more physical pain than those who slept 7 hours or more nightly (9.1%).

**Table:** 

Characteristic	Current Pain		
	n	N	% (95% Confidence Interval)
1–2 other chronic diseases	559	149,613	10.0 (8.7–11.5)
≥3 other chronic diseases	373	112,384	16.7 (14.0–19.9)
No other chronic disease	210	62,818	8.1 (6.2–10.6)
Binge drinking in past 30 days	90	26,923	10.0 (7.1–13.7)
No binge drinking in past 30 days	1,048	292,087	11.1 (9.9–12.1)
Current smoker	209	79,670	15.5 (11.8–20.0)
Not a current smoker	932	243,516	10.0 (9.0–11.1)
<7 hours of sleep per night	353	113,815	15.0 (12.3–18.2)
≥7 hours of sleep per night	569	142,927	9.1 (7.9–10.5)

**Figure 2:**
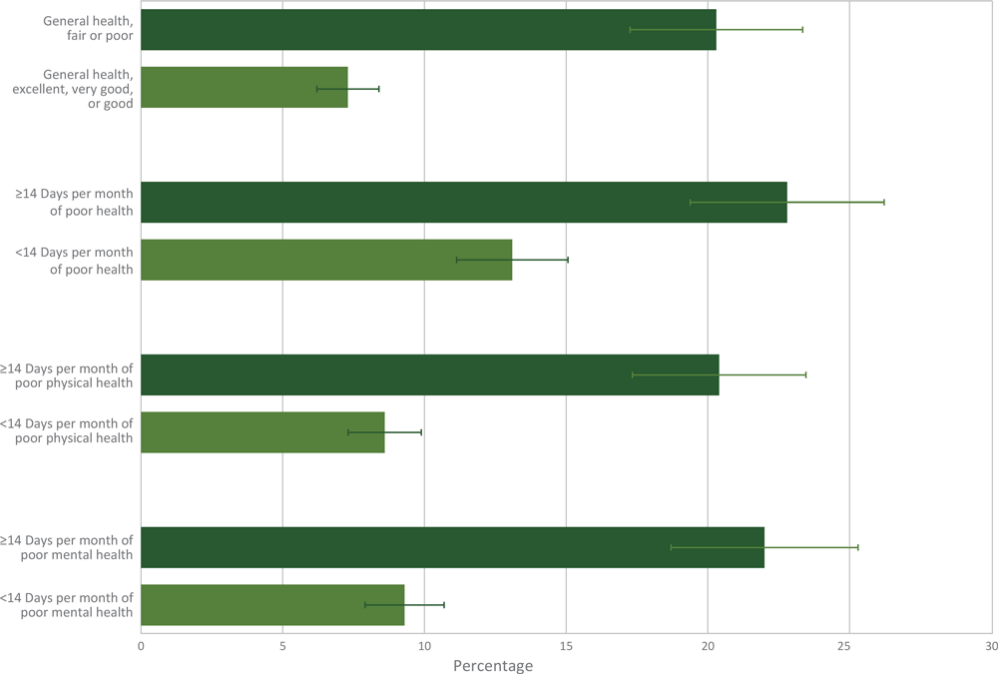
Percentage and 95% confidence intervals of current pain related to cancer or cancer treatment by physical and mental health characteristics. Brackets indicate confidence intervals.

The unadjusted prevalence of physical pain was higher among cancer survivors who reported fair or poor general health (20.3%) than among survivors who reported their general health as good, very good, or excellent (7.3%) (Figure 2). Physical pain was higher among cancer survivors who reported 14 or more days of poor general health (22.8%), poor physical health (20.4%), or poor mental health (22.0%) compared with survivors who reported less than 14 days of each (13.1%, 8.6%, and 9.3%, respectively). The adjusted prevalence of physical pain was higher among cancer survivors who reported fair or poor general health (18.0%), 14 or more days of poor physical health (16.6%), or 14 or more days of poor mental health (14.8%) compared with survivors reporting excellent, very good, or good health, or less than 14 days of poor physical or mental health (in the past 30 days) (Table 3).

**Table:** 

Characteristic	Unweightedn	WeightedN	Percentage Reporting Current Pain (95% Confidence Interval)
General health fair or poor	576	168,797	20.3 (17.7–23.2)
General health excellent, very good, or good	566	154,608	7.3 (6.3–8.5)
<14 days per month of poor mental health	876	232,927	9.3 (8.3–10.5)
≥14 days per month of poor mental health	253	87,729	22.0 (18.0–26.7)
<14 days per month of poor physical health	709	197,768	8.6 (7.5–9.9)
≥14 days per month of poor physical health	411	119,416	20.4 (17.6–23.6)
<14 days per month of poor health	563	162,144	13.1 (11.3–15.1)
≥14 days per month of poor health	283	89,903	22.8 (18.9–27.2)

**Table 3:** Adjusted Prevalence of Current Pain Related to Cancer or Cancer Treatment, by Overall, Physical, and Mental Health Among Cancer Survivors, Behavioral Risk Factor Surveillance System, 2012–2016

Variable	Current Pain
	% (95% CI)
≥14 poor physical health days^a^	16.6 (13.9–19.7)
<14 poor physical health days^a^	9.6 (8.3–11.0)
≥14 poor mental health days^b^	14.8 (11.8–18.5)
<14 poor mental health days^b^	10.3 (9.2–11.7)
Fair or poor health^c^	18.0 (15.5–20.8)
Excellent, very good, or good health^c^	8.3 (7.1–9.7)

Abbreviation: CI, confidence interval.

a Adjusted for race/ethnicity, age category, sex, educational status, marital status, employment status, insurance status, having multiple chronic diseases, and years since cancer diagnosis.

b Adjusted for race/ethnicity, age category, sex, educational status, marital status, employment status, insurance status, having multiple chronic diseases, and type of cancer.

c Adjusted for race/ethnicity, age category, sex, educational status, marital status, employment status, insurance status, having multiple chronic diseases, type of cancer, and years since diagnosis.

## Discussion

Cancer survivors commonly experience pain in the months and years after a cancer diagnosis (3,6–8). As cancer evolves into a chronic illness, so, too, may chronic pain. The characteristics associated with increased pain have population health implications that may help patients and providers to initiate collaborative discussions of optimal pain management. Pain that persists can have a negative effect on daily functioning and other possible negative outcomes (9,13,17), and patients deserve safe and effective pain management. Our findings indicate that from 2012 through 2016, nearly 10% of cancer survivors reported experiencing pain related to cancer or cancer treatment, and approximately 20% experiencing pain reported that pain was inadequately controlled.

Pain can be caused by the cancer itself, and those experiencing more advanced cancer are more likely to have pain (4,19). Pain can also be caused by cancer-related treatment or tests (4). Pain has been shown to be greatest among patients with certain cancers (14). Consistent with previous studies, our study found cancer-related pain to be highest among survivors of lung cancer, female breast cancer, leukemia/lymphoma, and colorectal cancer. Breast cancer survivors may experience increased pain related to tumor size, location, lymphedema, and potential spread to the nervous system, which can cause lingering neuropathic pain (16,19). Lung cancer survivors may experience increased pain at any cancer stage of development (ie, early or advanced). Such pain is usually of mixed pathophysiology, and a relatively high proportion is often attributed to neurologic damage from cancer treatment or metastasis to other organs (20). However, pain may also be increased among other cancer types that we were unable to examine individually in our study. Comprehensive assessment, including the effect of pain on function and quality of life, is important for all survivors (11), and long-term assessment can help to better recognize novel or previously unrecognized painful consequences of treatment (11). Effective pain management can generally be accomplished by regular screening (to recognize pain early), proper characterization of the pain (eg, acute or chronic, secondary to cancer, breakthrough), determination of optimal pharmacologic or nonpharmacologic treatment options, proper education for patients, and patient follow-up over time to titrate and adjust treatments (21). Useful clinical practice guidelines on unique considerations for use of opioids for pain control in cancer survivors are provided in the 2016 American Society of Clinical Oncology Clinical Practice Guideline on Management of Chronic Pain in Survivors of Adult Cancers and the 2018 National Comprehensive Cancer Network Clinical Practice Guideline for Prescribing Opioids for Chronic Pain (10).

Cancer-related pain control could be addressed early after a cancer diagnosis because inadequate control can lead to subsequent hospital visits and interference with daily activities (9). In our study, approximately 1 in 5 cancer survivors who experienced pain reported that it was inadequately controlled. Survivors reported pain related to cancer and cancer treatment for many years after the initial diagnosis. Telephone or web-based supportive care (22) or patient navigation (23) may help mitigate inadequate pain control. Public health programs can use these findings of characteristics associated with increased pain among cancer survivors to develop and implement interventions aimed at cancer survivors. The Centers for Disease Control and Prevention (CDC) National Comprehensive Cancer Control Program (NCCCP) awardees (ie, more than 60 state, territory, and tribal organization grant recipients) work in communities across the nation to prevent cancer, promote healthy lifestyles, and enhance cancer survivors’ quality of life (24). NCCCP awardees can seek partnership opportunities with health professionals to ensure that evidence-based guidelines and effective therapies are being applied. Given that studies have documented a high prevalence of inadequate pain control (12), physicians could consider including both short- and long-term pain management as part of cancer survivorship care plans and comprehensive palliative care strategies. Educating patients about how to routinely document and estimate pain between appointments may increase understanding of expected pain symptoms (15).

We found that cancer survivors who experienced pain were more likely to report a lack of sleep, comorbid chronic diseases, and smoking. Sleep disorders and mental health issues are common conditions in people with chronic pain (13,17,25), present in 17% and 90% of all adults in general, respectively (9). Many chronic pain syndromes include sleep disturbances, mood alterations, fatigue, and neurocognitive changes, which decrease quality of life (13,17). Chronic pain results in poor health-related quality of life among those with physical and mental health disorders (25,26). Cancer survivors in our study who reported pain were significantly more likely to report more than 14 days per month of poor general, physical, and mental health, which may lead to functional limitations (9). Given our findings and those of others, patients with multiple chronic conditions may require additional pain management strategies.

Ethnic and socio-demographic disparities are important considerations in care related to the prevalence, treatment, progression, and outcomes of pain management. The incidence and severity of chronic pain among cancer survivors has been shown to vary between racial and ethnic populations and by sex (13). Pain type, pain severity, number of pain locations, perceived etiology, pain interference, disability, functioning, and pain symptoms were all disproportionately higher among black respondents than among white respondents and among female respondents than among male respondents (13). Our findings were consistent with pain being more commonly reported among black cancer survivors than among white survivors and others and among female survivors. We also found that older cancer survivors (≥65) and cancer survivors who were first diagnosed when they were aged 65 or older reported cancer-related pain less. The most common types of cancer diagnosed among persons 65 or older were cancers of the lung, colon and rectum, stomach, breast, and prostate (2), but why people diagnosed at an older age were less likely to report pain is not entirely understood. A previous study of breast cancer survivors similarly found that younger age was associated with higher risk for chronic pain (27). These findings need to be interpreted cautiously because blacks in general (28) and elderly cancer patients (29) are at higher risk of undertreatment of chronic pain. Perceived discrimination and hopelessness have been implicated as reasons for this disparity among blacks (28); and elderly cancer patients are at higher risk for undertreatment because of assessment barriers such as memory or hearing loss, confusion, fear of being a burden, and stoicism (29).

Our study is subject to at least 5 limitations. First, BRFSS data are self-reported and are subject to the limitations associated with these types of data collection instruments, including respondent recall and social desirability bias. In particular, measures in the cancer survivor module to capture pain and pain management (eg, medication use and adherence) were brief and not as comprehensive as typical diagnostic pain assessment instruments used in clinical settings. A multidimensional pain instrument to capture pain severity, type, and functional impact would be preferable to characterize and inform pain management for cancer survivors (21). However, numerous studies have examined issues related to the reliability and validity of BRFSS and the system’s ability to provide valid estimates. Second, BRFSS provides limited information regarding respondents’ disease status (eg, disease-free, recurrence, end-of-life); we were only able to identify survivors currently being treated for cancer. Third, the cross-sectional nature of the data used for this study does not allow us to determine temporality for the relationships between pain and the factors examined. Fourth, BRFSS data on cancer-related pain were limited to the 18 states and territories that included the cancer survivor module during the years examined; these data are representative of only those survivors who responded to the survey in these states. Finally, it is possible that bias was introduced into these data because cancer survivors with the highest amount of pain may have been less willing or unable to participate in the survey.

Adequate palliative care for pain and symptom management through all phases of cancer treatment is a major concern for cancer survivors (30). Cancer survivors may not be aware how cancer-related pain may negatively affect their health and functioning in the years following treatment (9); thus, survivor characteristics associated with increased pain could help inform health care providers of cancer survivor populations that could most benefit from additional information to manage cancer-related chronic pain. The CDC NCCCP supports increasing knowledge of the most appropriate pain management planning and strategies for addressing short- and long-term chronic pain among cancer survivors (10,11,30). Partnerships between NCCCP cancer coalitions, health care providers, and treatment facilities may increase assessment, management, and planning for chronic pain among cancer survivors. Clinicians could also consider educating cancer survivors on available resources and strategies (21) to enhance informed decision making and alleviate patient fears related to pain medications (30).
